# Role of Myofibroblasts in the Repair of Iatrogenic Preterm Membranes Subjected to Mechanical Stimulation

**DOI:** 10.1002/pd.6722

**Published:** 2024-12-04

**Authors:** E. Costa, C. Thrasivoulou, D. L. Becker, J. Deprest, A. L. David, T. T. Chowdhury

**Affiliations:** ^1^ Centre for Bioengineering School of Engineering and Materials Science Queen Mary University of London London UK; ^2^ Department of Cell and Developmental Biology University College London London UK; ^3^ Lee Kong Chian School of Medicine Nanyang Technological University Singapore Singapore; ^4^ Department of Obstetrics and Gynecology University Hospitals Leuven Leuven Belgium; ^5^ EGA Institute for Women's Health University College London London UK

**Keywords:** Cx43, fetal membranes, fetal surgery, iatrogenic, PPROM, preterm birth

## Abstract

**Objective:**

We examined the role of myofibroblasts in regulating Cx43 and collagen structure in iatrogenic preterm amniotic membrane (AM) defects subjected to mechanical stimulation.

**Method:**

Preterm AM specimens were collected from women undergoing planned preterm caesarean section after in utero intervention for correction of spina bifida by open fetal surgery (*n* = 4 patients; preterm delivery at 34 + 0 weeks to 35 + 0 weeks). Control specimens taken 5 cm away from the open fetal surgery defect site were compared with wound edge AM. In separate experiments, the effects of mechanical stimulation and co‐treatment with Cx43 antisense on matrix and repair proteins were examined. Specimens were immunostained to detect αSMA and Cx43 in myofibroblasts and counterstained with DAPI to quantify nuclei shape. The direction of collagen fibrils in the wound edge region was examined by SHG imaging. Markers for matrix (collagen, elastin, GAG), inflammation (PGE_2_) and repair (TGFβ_1_) were examined by RT‐qPCR and biochemical assays.

**Results:**

In iatrogenic preterm AM specimens, the diameter of the open fetal surgery defect ranged between 3.5 and 7.5 cm. At the wound edge of the open fetal surgery defect, αSMA positive myofibroblasts had deformed nuclei and showed abundant Cx43 localized in the cell bodies or formed plaques. In the fibroblast layer, collagen had degenerated in some regions or had polarity near the wound edge. In preterm AM defects, mechanical stimulation and Cx43 antisense increased the levels of collagen and elastin but not GAG or PGE_2_ release. Mechanical stimulation increased Cx43 and TGFβ_1_ gene expression.

**Conclusion:**

In open fetal surgery defects, myofibroblasts were elongated with collagen fibrils that either degenerated or had polarity. Whilst cells produced substantially higher Cx43 in the fibroblast than in the epithelial layer, they formed plaques, which may prevent migration and delay healing. Mechanical stimulation of preterm AM enhanced matrix repair proteins and the mechanotransduction should be explored to understand how Cx43 contributes to membrane integrity.

**Summary**

- What is already known about this topic?
  ◦ Small defects < 3 mm in diameter do not heal after fetoscopic surgery and are prone to rupture.
  ◦ In preterm amniotic membrane (AM) defects created after fetoscopic surgery, amniotic mesenchymal cells (AMCs) form Cx43 plaques, which prevent migration and healing of the wound.
- What does this study add?
  ◦ In iatrogenic preterm AM with a defect size > 3.5 cm in diameter, myofibroblasts express αSMA and Cx43 in cell bodies and cytoplasmic processes.
  ◦ We also observed Cx43 plaques in the fibroblast layer near the wound edge, with regions of collagen degeneration around the defect site.
  ◦ In iatrogenic preterm AM explants subjected to mechanical stimulation, Cx43 antisense increased matrix and repair proteins.

## 1 Introduction

Strategies to seal and repair fetal membranes after prenatal interventions are important to prevent iatrogenic preterm premature rupture of fetal membranes (PPROM). After open fetal surgery, PPROM rates increase to between 30% and 45% of patients, largely due to the incision size, method of closure and poor healing mechanism [1, 2, 3]. Similarly, patients undergoing fetal laser surgery with small diameter fetoscopes result in tissue injury that can reduce biomechanical properties and weaken the membrane [4, 5, 6]. It is well established that the iatrogenic defect cannot heal and is more susceptible to amniotic fluid leakage and rupture [7, 8, 9, 10]. Currently, there are no sealing materials or clinical treatments to heal the wound and reduce PPROM after prenatal interventions.

Previous clinical studies have shown a varying ability of the fetal membranes to heal after trauma [11, 12, 13, 14, 15, 16, 17]. Several in vitro sealing techniques have been designed to control adhesion and improve biomechanics of the wound using patches, tissue adhesives, glues or plugs, but it remains unclear how these biomaterials would behave in a clinical setting [18, 19, 20, 21, 22, 23, 24, 25, 26, 27]. These approaches use in vitro inflation devices or animal models to test the mechanical strength and reported failure of the biomaterial due to poor structural properties, lack of adherence to the membrane defect, and incomplete membrane sealing leading to leakage of amniotic fluid [18, 19, 20, 21, 22, 25, 26, 27]. In human amniotic membrane (AM), amniotic mesenchymal stem cells (AMCs) have pluripotent properties and macrophages originating from the amniotic fluid have been shown to regenerate the AM and maintain mechanical elasticity after rupture [28, 29]. A combined tissue engineering approach to promote self‐healing at the site of injury could lead to better mechanical and adhesive properties after tensile or adhesive strength testing [30, 31]. However, regeneration in term AM was dependent on the size of the defect (1–3 mm), species (human, mouse, rabbits, ewe) and type of 3D model to investigate membrane healing after using sealants in culture for short term studies or in utero [7, 8, 21, 22, 23, 24, 25, 26, 27]. These findings demonstrate the complexity of the factors needed to design functional biomaterials for membrane wound healing and repair.

It is well established that partial healing in the AM increases proinflammatory cytokines such as interleukin‐1β (IL‐1β) and tumor necrosis factor α (TNFα), leading to matrixmetalloprotease‐9 (MMP‐9) and prostaglandin E2 (PGE2) production, which are associated with fetal membrane rupture [1, 6, 7]. Increased proteolysis degrades collagen at the defect site, compromising elasticity and mechanical strength, making the AM more prone to rupture [5, 10, 30, 31, 32, 33]. There is emerging evidence that in mouse AM with a small diameter (0.47 mm) but not a large defect (0.91 mm), embryonic wound healing by epithelial‐mesenchymal transition increased cell migration and collagen deposition enhanced by transforming growth factor β_1_ (TGFβ_1_) and cytokines [7]. We and others have shown that targeting connexin 43 (Cx43) with antisense in human term AM subjected to mechanical stimulation or trauma increased differentiation of AMCs into a myofibroblast population, coupled with matrix remodeling, inflammation, migration and repair [34, 35, 36, 37, 38, 39, 40, 41, 42, 43, 44]. However, the mechanism of mechanotransduction is dependent on the magnitude, duration and type of mechanical load applied in a static, dynamic, continuous or intermittent manner [34, 35, 36, 37, 38, 39, 41, 42, 44]. In iatrogenic injury with a large diameter defect created at the incision site during open fetal surgery, the mechanism of healing by myofibroblasts in the preterm AM are unknown. The present study examined myofibroblast behavior and collagen organization around the preterm AM defect site in patients undergoing prenatal spina bifida repair. We additionally explored whether mechanical stimulation and co‐treatment with Cx43 antisense could influence matrix and inflammatory proteins to enhance remodeling and repair mechanisms in preterm AM defects.

## 2 Methods

### 2.1 Research Ethics and Patient Recruitment

Human placentas (*n* = 4) were collected after planned caesarean section with written informed consent from women who delivered at University College London (UCL) Hospital (UCLH) following open fetal surgery in the second trimester. Ethical approval was granted by the Joint UCL and UCLH committees, the National Research Ethics Service Committee London, Bloomsbury and the Ethics of Human Research Central Office (Reference 14/LO/0863). All methods were performed according to the relevant guidelines and regulations at UCL, UCLH and Queen Mary University of London (QMUL).

### 2.2 Clinical Information

**FIGURE 1:**
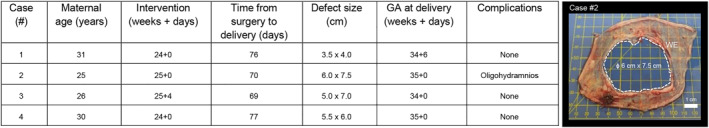
Clinical details for iatrogenic PPROM patients who underwent open fetal surgery for the correction of spina bifidae. Four cases of spontaneous ruptured fetal membranes were delivered preterm before 37 weeks of gestational age. The in utero intervention for correction of spina bifida neural tube defect by open fetal surgery took place between 24 + 0 and 25 + 0 weeks gestational age, creating a defect size between ∼3.0 and 7.5 cm, leading to late trimester iatrogenic preterm delivery between 34 + 0 and 35 + 0 weeks. In all cases, there was no infection after surgery or during the time up to delivery. All pregnancies were delivered by caesarean section. Image shows the size of a large diameter defect around 6 cm × 7.5 cm from a patient who delivered at 35 + 0 weeks complicated by oligohydramnios (Case #2). The approximate size of the defect is indicated for each case. Scale bar = 1 cm.

Open fetal surgical procedures for correction of open spina bifida in four patients and a photograph showing the size of the defect are presented in Figure 1. The in utero intervention took place between 24 + 0 weeks and 25 + 6 weeks of gestation leading to late trimester iatrogenic PPROM between 34 + 0 weeks and 35 + 0 weeks of gestation. Closure of the hysterotomy open fetal surgery site involved two layers of interrupted mattress sutures, which initially brought together the uterine incision. A further layer of interrupted sutures was placed at least 2 cm from the hysterotomy incision, incorporating the myometrium and fetal membranes to invert and thicken the hysterotomy closure site. None of the women experienced infection after open fetal surgery or during the 11 weeks leading to caesarean section delivery. Women with multiple pregnancies were excluded from this study.

### 2.3 Isolation of Preterm Fetal Membrane Defect and Culture

In all open fetal surgery cases, the delivery of the fetus was via routine entry at caesarean section through the lower segment of the uterus. The hysterotomy wound from the open fetal surgery was located on the uterus and checked for integrity. The orientation of the membrane to the placenta, caesarean section incision line and cervix was noted throughout the procedure and the fetal membranes nearest the cervix were identified using a clip. The placenta and fetal membranes were then delivered by controlled cord traction and examined to identify the open fetal surgery defect in the fetal membranes. A control region in the fetal membrane that was aligned in the same axis as the open fetal surgery defect and was at least 5 cm away from the wound edge was excised along with 2 cm margins of the full thickness membrane. Control and wound edge AM specimens near the open fetal surgery defect site were immediately fixed in 4% PFA for 2 h and stored in PBS prior to immunostaining. In addition, the AM was separated from the CM and placenta using gentle traction and washed with Earle's Balanced Salt Solution for 2 min (Sigma‐Aldrich, UK). Dumbbell shaped AM specimens measuring widths in the gauge and shoulder regions of 5 mm × 10 mm were dissected and explants equilibrated in 1 mL of Dulbecco's modified Eagle's medium (DMEM) supplemented with 5 μg/mL penicillin, 5 μg/mL streptomycin, 15 μg/mL ascorbate and 20% fetal calf serum (FCS) prior to mechanical loading experiments.

### 2.4 Effect of Mechanical Loading

Human preterm AM specimens were secured within a Bose loading frame (Bose Corporation, Eden, Prairie, Minnesota, USA) at 37°C. Serum free media was supplemented with 0 or 50 μM Cx43 antisense (inhibits Cx43 mRNA expression). Strained preterm AM specimens were subjected to 2% CTS at 1 Hz frequency applied intermittently (1 min CTS followed by 9 min unstrained) for 4 and 24 h, as described [42, 44]. In separate experiments, a 0.8‐mm defect was created with a 21G needle in the explant to mimic trauma and preterm AM explants were subjected to CTS. In control experiments, specimens were taken from the same donor and cultured without mechanical stimulation (−CTS).

### 2.5 Tissue Preparation for Immunostaining

AM specimens were incubated with primary antibodies for mouse Cx43 (1:100, ThermoFisher Scientific, CX‐1B1) and rabbit αSMA (1:100, Abcam, ab5694) at 4°C overnight as described 44 Specimens were washed in PBS and incubated with secondary antibodies for Alexa Fluor 568 anti‐mouse or 488 anti‐rabbit at room temperature for 2 h (both 1:1000, ThermoFisher Scientific) and counterstained with 1 μg/mL DAPI for 20 min (1:1000) to detect nuclei. Secondary antibody incubation in the absence of the primary antibody was used as a negative control.

### 2.6 Second Harmonic Generation and Confocal Imaging

Specimens from open fetal surgerg were imaged by two‐photon confocal imaging on a Leica TCS SP8 acousto‐optic beam splitter multiphoton confocal laser scanning microscope (Leica, Milton Keynes, UK) with a Coherent Chameleon Ultra, Ti Sapphire mode locked IR laser (Coherent UK Ltd, Cambridge, UK). Samples were imaged at excitation/emission wavelengths of 405/460 nm for DAPI, 495/518 nm for αSMA and 578/603 nm for Cx43, as previously described [41, 42, 43, 44]. A transmission detector was used for the collection of collagen SHG signal with a 430–450 nm barrier filter with a pump wavelength of 880 nm at 80 fs pulse width. A constant step size Z‐section interval of 1.5 μm was used across all Z‐stack images collected. All parameters including detector gain, offset and laser power were kept constant to enable quantification. Images were processed using ImageJ software (version windows 64 bit, Fiji) and Imaris 9.5.0. High resolution images were taken on the Zeiss LSM 980 with Airyscan 2 microscope (Zeiss, Oberkochen, Germany) with ×40 magnification and identical excitation/emission wavelengths for DAPI and αSMA. Multiple tile images were taken from a rupture area of 2–5 mm and stitched together using the pairwise stitching command on ImageJ (Fiji). Control images were taken approximately 500 μ away from the rupture site.

### 2.7 Confocal Image Quantification

The immunostaining confocal imaging technique was used to detect αSMA expressed by myofibroblasts with an antibody that recognized the Cx43 molecule. Cx43 levels were quantitatively evaluated per tissue area and per cell nuclei in preterm AM specimens using a well‐established pixel‐counting method, as previously described [42, 43]. Maximum projections were performed in the AM to detect Cx43 levels expressed by cells present in the epithelial and fibroblast layers. The images were converted to binary using identical threshold values and objects exceeding 2 pixels were counted to identify Cx43‐positive pixels per tissue area (500 μm^2^) or per cell nuclei. To quantify nuclei roundness, maximum RGB projections of the DAPI signal were converted to binary and using the particle measurement feature in ImageJ (Fiji) software, circularity values of nuclei defined by 4π × area/perimeter^2^ were calculated. Nuclear circularity analysis shows that a value of 1 indicates a perfect circle and reduces when the nucleus becomes increasingly elongated. To determine the direction of collagen alignment, SHG images were converted to binary and the 2D orientation analysis was performed using the local gradient orientation method and directionality ImageJ plug‐in (v2). This software calculates the amount of objects that are distributed between either 0° and 180° or −90° and 90°, depending on the image direction of collagen fibers with a bin size of 1°.

### 2.8 Biochemical Analysis

DNA, GAG and collagen concentrations were measured by Hoechst 33258, 1,9‐dimethylmethylene blue and hydroxyproline assays (all reagents from Sigma‐Aldrich Chemical Company, Poole, UK). Elastin concentration was determined by the fastin elastin dye‐binding method (Biocolor Life Science Assays, Co Antrim, UK). PGE2 release was determined by immunoassay (R&D Systems, Europe Ltd, Abingdon).

### 2.9 Quantitative PCR

The methods for RNA extraction, cDNA synthesis and RT‐qPCR have been described 43–44. Primer pair sequences for Cx43 sense: 5′‐CTCGCCTATGTCTCCTCCTG‐3′, antisense: 5′‐ TTGCTCACTTGCTTGCTTGT‐3′, TGFβ1 sense: 5′‐CCCAGCATCTGCAAAGCTC‐3′ and antisense: 5′‐GTCAATGTACAGCTGCCGCA‐3′ and GAPDH sense: 5′‐ TCTCTGCTCCTCCTGTTC‐3′, GAPDH antisense: 5′‐CGCCCAATACGACCAAAT‐3′. PCR products were detected on the StepOnePlus Real‐Time PCR System (ThermoFisher Scientific). Relative gene quantification of Cx43 or TGFβ_1_ was calculated by normalizing the target to the reference gene GAPDH and to the calibrator sample by a comparative Ct approach and ratio values expressed on a logarithmic scale.

### 2.10 Statistical Analysis

For all experiments, the number of replicate specimens examined for each test per donor is indicated in the figure legend. All values are expressed as the mean and ±SEM. Statistical comparisons for the multiple groups were performed using a post hoc Bonferroni‐corrected *t*‐test where values of *p* < 0.05 were considered statistically significant.

## 3 Results

### 3.1 Myofibroblast Morphology in Iatrogenic Preterm AM

**FIGURE 2:**
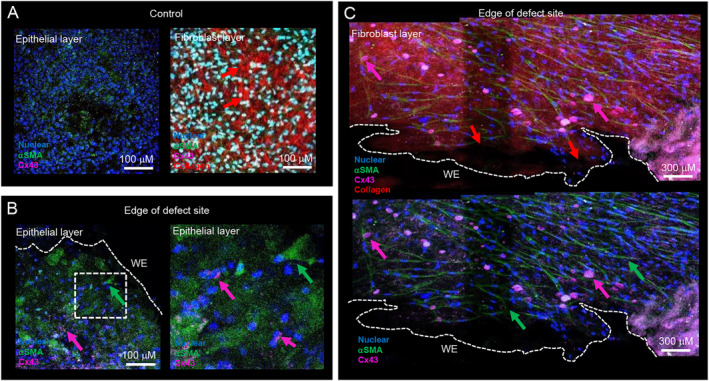
Myofibroblast morphology in human iatrogenic preterm AM. Fetal membranes were taken from a 31 year old patient who underwent open fetal surgery for correction of spina bifida neural tube defect that took place at 24 + 0 weeks gestational age leading to late trimester preterm delivery at 34 + 6 weeks (Case #1). Myofibroblast morphology was examined in αSMA expressing cells (green arrow) by IMF confocal microscopy in control AM specimens (A) and near the edge of the defect site (B, C). Cx43 was detected by immunostaining (pink arrow, C) and collagen by SHG imaging (red arrow). Control AM specimens were taken 5 cm away from the defect site. Signals for blue (DAPI), green (αSMA), pink (Cx43) and red (collagen) were detected by IMF confocal microscopy and SHG imaging. The dotted white lines show the length of the wound edge (WE) in the iatrogenic preterm AM specimen. Sale bar = 100 μM (A, B) and 300 μM (C). The white dotted box in (B) shows an enlarged image to show Cx43 localized in cell bodies.

Figure 2 shows the edge of the AM open fetal surgery defect site from a 31‐year‐old patient who underwent open fetal surgery for correction of spina bifida that took place at 24 + 0 weeks gestational age leading to late trimester preterm delivery at 34 + 6 weeks. In the epithelial layer of control preterm AM, we observed the presence of myofibroblasts that stained positive for αSMA by IMF confocal microscopy (Figure 2A). In the fibroblast layer, there was increased αSMA expressing myofibroblasts and collagen fibers had shorter basket shaped fibers organized in a random fashion (red arrow, Figure 2A). At the edge of the open fetal surgery defect site, αSMA positive myofibroblasts expressed cytoplasmic Cx43 in the epithelial layer (pink arrow, Figure 2B) and formed Cx43 plaques in the fibroblast layer (pink arrow, Figure 2C) near the edge of the open fetal surgery defect site. We observed an absence of collagen near the wound edge of the open fetal surgery defect site (red arrow, Figure 2C), but there were regions of collagen in the fibroblast layer.

### 3.2 Nuclei Morphology and Cx43 Plaque Formation in Iatrogenic Preterm AM

**FIGURE 3:**
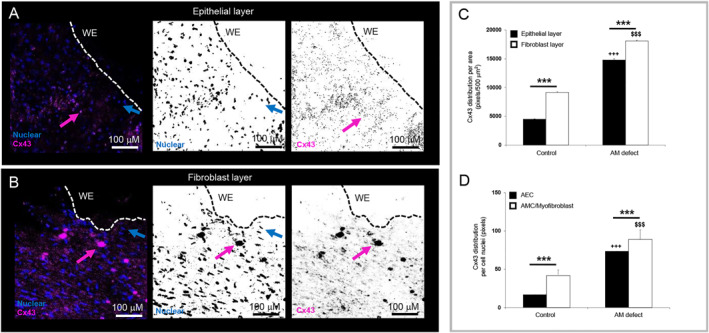
Cx43 plaque formation in human iatrogenic preterm ruptured amniotic membrane. Representative images obtained by confocal microscopy are shown in the epithelial (A) and fibroblast layer (B) of the preterm AM defect from one late third trimester iatrogenic preterm donor (Case #1). The distribution of Cx43 was analyzed per tissue area for comparisons between the epithelial and fibroblast layers (C) or per cell nucleus (D). Error bars represent the mean and SEM values of 6 replicates from three late trimester iatrogenic preterm donors (34 + 0 weeks to 35 + 0 weeks, Case #1–3). Significant differences are indicated by ***p < 0.001. Statistical comparisons are indicated for control AM specimens and AM defect (*p* < 0.001***) in the epithelial layer (*p* < 0.001^+++^) and fibroblast layer (*p* < 0.001^$$$^). The dotted white and black lines show the length of the wound edge (WE) in the preterm AM specimen. Sale bar = 100 μM (A, B).

Representative images obtained by confocal microscopy are shown from one late third trimester iatrogenic preterm donor (Figure 3). In the epithelial layer, Cx43 was expressed by AECs present at the wound edge of the open fetal surgery defect site (Figure 3A) and in the fibroblast layer, had formed large plaques (Figure 3B, pink arrows). We next compared Cx43 distribution expressed by cells present in the epithelial and fibroblast layer of AM defect (Figure 3C,D). The levels of Cx43 protein expression were significantly increased compared with control specimens (*p* < 0.001; Figure 3C). The nuclei of all cell types expressed Cx43 in control AM (AEC 17.1 pixels; AMC/myofibroblast 41.8 pixels) and the values significantly increased in the AM defect (73.7 and 89.2 pixels, respectively; both *p* < 0.001; Figure 3D).

### 3.3 Myofibroblast Nuclei Deformation in Human Iatrogenic Preterm AM

**FIGURE 4:**
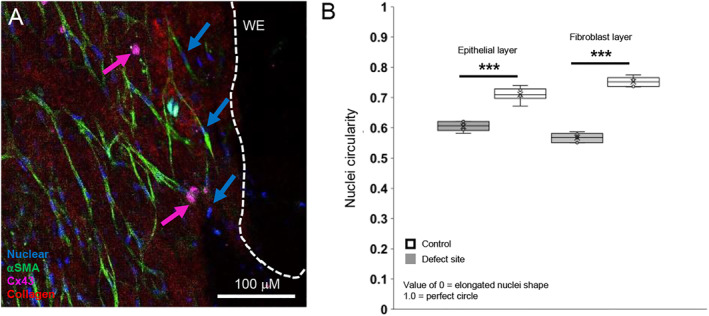
Myofibroblast nuclei deformation in human iatrogenic preterm AM. AM specimens were immunostained for αSMA to detect myofibroblast (pink arrow) nuclei (blue arrow, A). Circularity values for the nuclei shape of myofibroblasts expressing αSMA in the epithelial layer were compared to the fibroblast layer in control specimens and AM defect (B). Nuclei values close to 1 represent a perfect circle in contrast to zero, which represents a more elongated shape. The total number of nuclei counted ranged from 1202 to 1582 from 6 specimens (Case #1–3). Signals for blue (DAPI), green (αSMA) and pink (Cx43) were detected by immunofluorescence confocal microscopy. The dotted white lines show the length of the wound edge (WE) in the iatrogenic preterm AM specimen. Scale bar = 100 μM. ***p < 0.001.

Immunostaining for αSMA detected highly elongated myofibroblasts (pink arrow) with elongated nuclei near the wound edge of the open fetal surgery defect site (blue arrow, Figure 4A). 3D sliced imaging of the AM open fetal surgery defect site showed the absence of cells and collagen near the wound edge (Figure 4A). Circularity values for the nuclei shape of myofibroblasts expressing αSMA present in the epithelial layer were compared to the fibroblast layer in control specimens and AM open fetal surgery defect (Figure 4B). Values for nuclei shape for AECs and AMCs/MFs present in control AM specimens ranged from 0.71 (±0.006) to 0.75 (±0.006) in contrast to AMC/myofibroblast with values which are broadly similar in the epithelial (0.60 ± 0.009) and fibroblast layer (0.56 ± 0.005).

### 3.4 Collagen Organization in Iatrogenic Preterm AM

**FIGURE 5:**
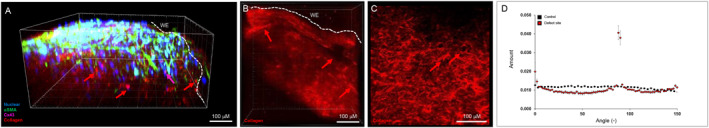
Collagen organization in human iatrogenic preterm ruptured amniotic membrane. SHG imaging at the defect site showed the presence of a dense region of collagen fibers which either formed clusters (red arrow, A) or had alignment tangential to the wound edge (B) or were absent and had degenerated (red arrow, B). The organization of collagen in a basket‐like fashion in control preterm AM are shown in (C). Analysis of the direction of collagen fibers at a specific polarization angle is shown in the alignment graph (D). Error bars represent analysis of collagen fiber alignment in three regions and analysis repeated with six replicates taken from three late third trimester iatrogenic preterm donors (34 + 0 weeks to 35 + 0 weeks, Case #2–4). The dotted white lines show the length of the wound edge (WE) in the iatrogenic preterm AM specimen. Representative SHG images are shown for one field of view, where scale bar = 50 μm.

Combined 3D sliced confocal and SHG imaging showed the presence of dense regions of collagen fibers that appeared shorter and had formed clusters in the fibroblast layer of the preterm AM open fetal surgery defect (red arrow, Figure 5A). Some of the fibers were aligned tangential to the open fetal surgery defect site but had also degenerated near the wound edge (red arrows, Figure 5B) in contrast to basket‐like fibers that appeared in a random fashion in control specimens (red arrows, Figure 5C). Analysis of the directionality of collagen showed that some fibers had a clear polarization angle at around 88 ° and 90, while in other regions near the wound edge of the open fetal surgery defect, the fibers were disorganized (Figure 5D).

### 3.5 Effects of Mechanical Stimulation on Protein and Gene expression

**FIGURE 6:**
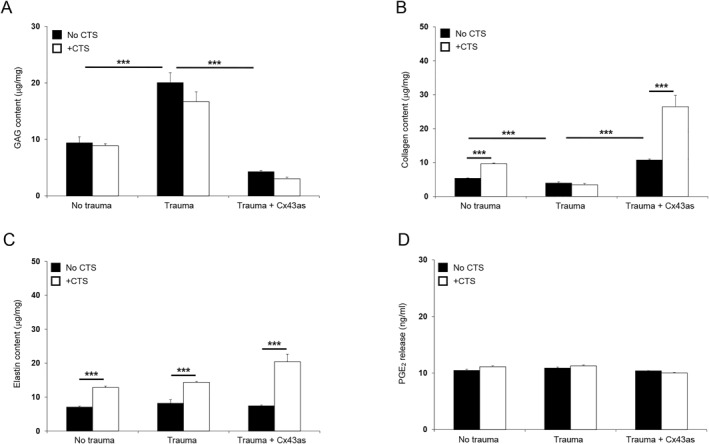
Effects of mechanical stimulation in human iatrogenic preterm AM. Preterm AM specimens were traumatized with a needed to create a 0.8‐mm defect and subjected to cyclic tensile strain (CTS) for 24 h. Mechanical stimulation was applied intermittently at 2% strain and 1 Hz frequency in the presence and absence of 50 μM Cx43 antisense (Cx43as). Absolute values for GAG (A), collagen (B) and elastin content (C) were normalized to dry tissue weight. PGE_2_ release was quantified in media samples (D). Explants cultured without cyclic tensile strain (−CTS) were compared to +CTS specimens. Error bars represent the mean and SEM values of 24 replicates from three late third trimester iatrogenic preterm donors (34 + 0 weeks to 35 + 0 weeks, Case #1–3). Significant comparisons are indicated for −CTS and +CTS conditions where ***p < 0.001. All other comparisons (not indicated) were not significantly different.

**FIGURE 7:**
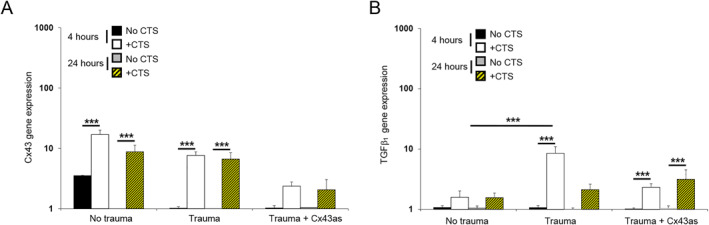
Effects of mechanical stimulation on gene expression in human iatrogenic preterm AM. Preterm AM specimens were traumatized with a needed to create a 0.8‐mm defect and subjected to cyclic tensile strain (+CTS) for 4 and 24 h. Mechanical stimulation was applied intermittently at 2% strain and 1 Hz frequency in the presence and absence of 50 μM Cx43 antisense (Cx43as). Gene expression of Cx43 (A) and TGFβ (B) are presented as ratio values and normalized to control values. In all cases, error bars represent the mean and SEM values of 12 replicates from three late third trimester iatrogenic preterm donors (34 + 0 weeks to 35 + 0 weeks, Case #1–3). Significant comparisons are indicated for −CTS and +CTS conditions where ***p < 0.001. All other comparisons (not indicated) were not significantly different.

Figure 6 examined absolute values for GAG, collagen, elastin and PGE_2_ release from iatrogenic PPROM donors who delivered late preterm. After 24 h of mechanical stimulation, CTS significantly increased GAG content in AM after trauma (*p* < 0.001) and this response was reduced by the presence of the Cx43 antisense (*p* < 0.001; Figure 6A). In control but not trauma AM specimens, CTS increased collagen content and this response was significantly enhanced by co‐stimulation with Cx43 antisense (all *p* < 0.001; Figure 6B). In control and trauma AM specimens, absolute values for elastin content were broadly similar, but co‐stimulation with Cx43 antisense increased elastin (*p* < 0.001, Figure 6C). Additionally, values for PGE_2_ release ranged from 10.0 to 11.2 ng/mL in all test conditions examined and were not influenced by CTS or co‐treatment with the antisense (Figure 6D). Figure 7 examined the effects of mechanical stimulation on Cx43 and TGFβ_1_ gene expression in preterm AM defects after 4 and 24 h of culture. At each time point, CTS significantly increased Cx43 gene expression in control AM and after trauma and this response was reduced with the antisense (*p* < 0.001, Figure 7A). Co‐stimulation by CTS and Cx43 antisense significantly increased TGFβ gene expression in AM defect specimens (*p* < 0.001; Figure 7B).

## 4 Discussion

In preterm open fetal surgery for AM defects, we confirmed the presence of myofibroblasts that express αSMA and Cx43 localized in the cell bodies and cytoplasmic processes. Whilst AMCs have been reported to differentiate into myofibroblasts during inflammation and wound healing, the expression of αSMA incorporated into stress fibers is a marker that classifies these cells and functions to transmit contractile forces, increasing stiffness, TGFβ release and collagen for wound contraction activated during the early phase of the healing in several tissue types [45, 46, 47, 48, 49, 50]. Transmission of this contractile force is through the extracellular matrix and it has been shown that αSMA increased contractility of myofibroblasts by modifying myosin stress fibers and cell traction forces generated through actomyosin filament sliding [51, 52]. This process allows myofibroblasts to move through the collagen rich matrix via focal adhesions pulling the wound edges together in a purse‐string like manner enabling contraction of collagen at the wound margin [51, 52, 53]. Cx43 activates myofibroblast migration, possibly through pro‐inflammatory macrophage‐activated TGFβ_1_ signaling and N‐cadherin to aid migration and subsequent αSMA filament sliding [54, 55, 56, 57, 58]. Nevertheless, large iatrogenic open fetal surgery defects more than 3 cm cannot undergo purse‐string wound contraction that is seen in embryonic wound healing but might after suturing [59]. Indeed, in the myometrium of iatrogenic membrane defects, there was enhanced CD68+ macrophage infiltration and healing of membranes in mice compared with clinically [7, 60]. However, membrane integrity and cell mechanics are enhanced by the ability of collagen to polarize and contract the edges of the wound [55, 56]. This purse‐string contraction mechanism of healing may be lost, which is why there is no spontaneous healing of large membrane defects. It is not clear how defect size when using large diameter fetoscopes with multiple ports plays a role in membrane healing, thereby increasing the risk of PPROM [9].

In the present study, iatrogenic preterm open fetal surgery AM defects had a highly contractile myofibroblast population with elongated nuclei, cell bodies and cytoplasmic extensions, but the collagen fibers appeared either to have degenerated or had formed clusters at the wound edge. We and others have shown that collagen structure in preterm AM defects after laser surgery for TTTS treatment was abundant with a loss of normal fibril pattern and an increased number of distorted whorl‐like fibrils creating a thickened wound edge similar to previous studies [20, 28, 56]. In contrast, collagen concentration increased at the defect and suture site in fetal membranes after SBA open fetal surgery, with region dependent gene expression of MMP‐1/9 and TIMP‐1/2 [8]. In our study, collagen density and abnormal fiber orientation formed collagen clusters coupled with increased apoptosis near the edge of the preterm AM defect site, where we also observed no nuclei staining. It is not clear if the myofibroblast population generated through AEC/AMC differentiation as found in other tissues causes cells to move through the matrix layers via focal adhesions while transition in the fibroblast layer remains compromised leading to apoptosis, as reported by others in term membranes or primary human AMC culture [47, 55, 61]. Cyclic EMT‐MET transition is well known to enhance tissue integrity throughout gestation, while favorable EMT‐MET differentiation increases towards term and preterm inflammation or TGFβ_1_ suppressed EMT transcription factors and maintains AEC morphology [61]. Whilst we did not examine apoptosis or cell senescence, EMT inflammation degrades collagen by extracellular vesicles, which carry inflammatory factors such as MMPs from the myometrium to the fetal membranes [6, 32]. Proteomic analysis of inflammatory exosomes identified several inflammatory pathways which activate TGFβ_1_, COX‐2 or MMP‐9 or TNFα in a variety of membrane tissues and models [57]. These studies highlight the multifactorial mechanisms that lead to AEC/AMC differentiation of cell phenotype leading to an increase of myofibroblast population and the need to use EMT‐MET markers, particularly E‐cadherin, to define the mechanism of healing.

In the mechanotransduction studies, we see drastic changes in matrix protein levels after trauma since the membranes are from open fetal surgery for correction of spina bifida. There is a two‐layer closure of the myometrium and fetal membranes after fetal surgery to prevent chorioamniotic membrane separation (CMS) and related PPROM. To ensure a tight hysterotomy closure, the surgeons performed two layers of interrupted mattress sutures, which initially brought together the hysterotomy incision. Then, a further layer of interrupted sutures placed at least 2 cm from the hysterotomy incision and incorporating the myometrium and fetal membranes is used to invert and thicken the hysterotomy closure site. This results in trauma not only at the site of the open fetal surgery incision but also at least 2 cm away from it. Furthermore, there is likely to be hemorrhage and pro‐inflammatory pathology occurring beyond the suture area during healing. This is likely to result in inflammation being present beyond 5 cm of the open fetal surgery defect, which was the region of the fetal membranes sampled in this study. We therefore hypothesize that there is a “field effect” leading to a spatial/temporal cascade of catabolic and pro‐inflammatory processes that ultimately degrade the tissue. Importantly, myofibroblasts are mechanosensitive cells that can detect changes in tissue stiffness by activating repair pathways involving TGFβ and collagen release. However, there is a lack of understanding of how the mechanical environment affects the pathways activated by myofibroblasts to promote stiffness, ECM changes and structural integrity and cross talk with signaling pathways after diagnosis for fetal growth restriction, spontaneous or iatrogenic preterm birth deliveries. Whilst stretching of term membrane explants increased pro‐inflammatory cytokines, PGE_2_ and collagenases, mechanotransduction pathways in preterm membranes are not understood [62, 63, 64, 65]. Cx43 is also a mechanosensitive protein that can either hinder migration or upregulate transcriptional activity, including proliferation, migration and differentiation, as previously reported in terms of AM and skin wounds [40, 43, 48, 49, 50, 51]. In the myometrium, Cx43 is activated by mechanical stretch, resulting in gap junctions between myometrial smooth muscle cells for synchronization of contraction, migration and healing [38]. Whilst prostaglandins can regulate Cx43 expression and increase the risk of preterm labor, the high PGE_2_ levels found in preterm membranes (> 10 ng/mL) compared to term (< 2.5 ng/mL) may indicate activation of the uterine contraction phenotype resulting in early cervical ripening possible due to increased GAG levels [66, 67] Taken together, future studies should examine the mechanotransduction mechanisms in preterm membranes.

## 5 Conclusions

Large iatrogenic open fetal surgery AM defects do not heal after rupture. Whilst cells formed Cx43 plaques near the defect site, myofibroblasts express αSMA and appear to be highly elongated and contractile. The αSMA population was localized around the wound edge of the open fetal surgery defect, characterized by regions of collagen that had polarity or had degenerated near the edge of the open fetal surgery defect. It is not clear whether activation of myofibroblasts is essential for healing and repair of membranes. After mechanical stimulation of iatrogenic preterm open fetal surgery AM defects, knockdown of Cx43 with antisense increases matrix proteins and TGFβ. Inflammation induced by open fetal surgery or the mechanical environment should be investigated with human in vitro biomechanical models to determine the mechanisms after injury and pathological changes characterized by inflammation. Alterations in the subcellular localization of Cx43 may be associated with morphological changes in myofibroblasts and mechanotransduction signaling. Regulation of these mechanisms will be important for therapeutic strategies aimed at reducing the risk of rupture, subsequent infection and preterm birth.

## Ethics Statement

Ethical approval for fetal membrane sample collection was granted by the Joint UCL and UCLH Committees on August 15, 2014 (REC reference: 14/LO/0863).

## Conflicts of Interest

The authors declare no conflicts of interest.

## Data Availability

The data that support the findings of this study are available from the corresponding author upon request.
